# Mechanisms of Exercise-Mediated Regulation of the Gut–Brain Axis in Parkinson’s Disease

**DOI:** 10.3390/nu18101639

**Published:** 2026-05-21

**Authors:** Xiaofan Men, Wei Wu

**Affiliations:** School of Athletic Performance, Shanghai University of Sports, Shanghai 200438, China; menxiaofan@sus.edu.cn

**Keywords:** Parkinson’s disease, gut microbiota, gut–brain axis, exercise, diet

## Abstract

Parkinson’s disease (PD) is a progressive neurodegenerative disorder that is closely associated with dysfunction of the gut–brain axis. Exercise and diet exert neuroprotective effects on PD by regulating the gut–brain axis, yet the overall mechanisms underlying this regulation remain to be systematically elucidated. This article reviews the characteristic changes in gut microbiota during the progression of PD and the pathological mechanisms involving gut–brain axis dysfunction. It systematically outlines the intrinsic mechanisms by which gut microbiota modulate the onset and development of PD from the perspectives of metabolism, immunity and inflammation, neuroendocrinology, and the temporal and causal relationships between gut microbiota and PD. On this basis, the discussion focuses on the regulation of the gut–brain axis through exercise to improve PD, with emphasis on remodelling the composition and diversity of gut microbiota, enhancing gut barrier and blood–brain barrier (BBB) functions, regulating immune and inflammatory homeostasis, upregulating the expression of neurotrophic factors and promoting neuroplasticity, as well as the synergistic effects of exercise and diet. In parallel, the independent and synergistic effects of dietary interventions (e.g., high-fibre and Mediterranean diets) are discussed. In addition, the effects of different types of exercise on alleviating PD by regulating gut–brain axis are analysed. This review aims to provide new insights and a scientific basis for the prevention and intervention of PD.

## 1. Introduction

Parkinson’s disease PD is the second most common neurodegenerative disorder worldwide. Its main clinical features include motor symptoms, such as bradykinesia and resting tremor, as well as non-motor symptoms, including constipation and depression [1]. With the acceleration of global population ageing, the disease burden of PD is rising at an unprecedented rate. There are currently approximately 10 million patients worldwide, and this number is projected to reach 17 million by 2040 [2,3]. This has become a major public health crisis that seriously affects the quality of life of older adults. In China, population ageing has further intensified the disease burden of PD, posing serious challenges to family care and the healthcare system [4].

Current treatment for PD is primarily based on dopaminergic replacement therapy, such as levodopa. However, long-term use is often associated with serious limitations, including reduced efficacy and motor complications [5]. Therefore, exploring non-pharmacological strategies that can delay disease progression and improve quality of life has become a research priority. In recent years, the central role of the gut–brain axis in the pathogenesis of PD has become increasingly evident [6]. The Braak hypothesis, which proposes that PD pathology may originate in the gut and spread to the brain via the vagus nerve, has provided a groundbreaking framework for understanding disease initiation [7]. This framework is supported by clinical evidence showing that gut microbiota dysbiosis is common in PD patients and closely associated with alpha-synuclein (*α-syn*) aggregation, neuroinflammation, and BBB disruption [8]. These findings suggest that targeting the gut microbiota and its communication with the central nervous system may represent a new approach to intervening in the progression of PD.

Although existing reviews have separately described the association between gut microbiota and PD, or the beneficial effects of exercise on PD, a systematic framework that integrates how exercise regulates the course of PD specifically through the gut–brain axis remains incomplete [9]. A recent bibliometric analysis has comprehensively mapped the research landscape of exercise, the gut–brain axis, and cognitive impairment in PD [10]. The present review differs in that it focuses on mechanistic comparisons across exercise modalities and on synergistic effects with diet. Different exercise modalities such as aerobic, resistance, and mind–body exercises may exert distinct regulatory effects on the gut–brain axis. At present, there is a lack of critical comparison of these differences [11]. Mind–body exercises, represented by Tai Chi, possess the dual characteristics of peripheral muscle activity and central nervous system regulation. As such, they may exert a unique modulatory effect on the gut–brain axis by optimising autonomic balance, for example, by enhancing vagal tone [12,13]. However, this potential remains largely unexplored.

Dietary patterns independently shape gut microbiota composition and function. The Mediterranean diet is associated with reduced PD risk and lower pro-inflammatory cytokine levels [14,15]. Dietary fibre serves as a substrate for Short-Chain Fatty Acids (SCFA) producing bacteria, reinforcing intestinal barrier integrity and modulating systemic inflammation. However, few reviews have systematically integrated the interactive effects of exercise and diet [16,17]. This review, while focusing on the mechanisms of exercise, will also explore the potential synergistic effects of exercise and diet—such as high-fibre and Mediterranean diets—and provide a theoretical basis for multidimensional lifestyle interventions.

## 2. Overview of PD

The typical pathological features of PD are the loss of dopaminergic neurons in the substantia nigra and the formation of Lewy bodies, which result from the abnormal aggregation of *α-syn*. Non-motor symptoms, such as constipation, often manifest several years or even decades before the onset of motor symptoms [18]. PD is caused by the complex interaction between genetic susceptibility and environmental factors [19]. Most sporadic cases are believed to be related to dietary patterns, physical activity levels, and exposure to environmental toxins, and are closely related to modifiable lifestyle factors [20]. This provides a theoretical basis for lifestyle interventions such as exercise.

From a pathogenic perspective, although the traditional view focuses on intracellular events such as mitochondrial dysfunction, oxidative stress, and neuroinflammation, these processes may not be the initiating steps in the pathological process [21]. Currently, dysfunction of the gut–brain axis is considered a key upstream event driving the pathological progression of PD. Disruption of the gut microbiota increases intestinal barrier permeability, a condition known as “leaky gut”. This allows bacterial metabolites and toxins to enter the bloodstream, thereby triggering systemic inflammation and disrupting the integrity of the BBB. This results in *α-syn* aggregation, microglial cell activation, and degeneration of dopaminergic neurons in the central nervous system [22]. This intestinal-brain cascade provides a reasonable explanation for the early appearance of non-motor symptoms and the multisystem nature of the disease.

The current focus of PD treatment is to improve motor symptoms through drugs such as Levodopa. However, this approach does not prevent the progression of the disease, and long-term use often comes with challenges such as motor complications [23]. This has prompted researchers to turn to interventions with neuroprotective potential and sustainability. Recent evidence indicates that the neuroprotective effect of exercise may not be mediated by a single pathway [24]. It is partly due to regulation of the gut–brain axis, in which exercise remodels the gut microbiota and enhances barrier function, thereby reducing neuroinflammation, promoting the expression of neurotrophic factors, and ultimately slowing neurodegenerative changes [25]. This updated understanding opens up new avenues for comprehensive PD intervention, that is, acting on the brain to affect the gut.

## 3. Gut Microbiota, the Gut–Brain Axis, and PD

### 3.1. Gut Microbiota Alterations in Patients with PD

A large body of research has confirmed that the composition of the gut microbiota in patients with PD undergoes profound changes [26]. This dysbiosis is considered a key component in the pathogenesis of PD. Overall, the gut microbiota in patients with PD is characterised by the coexistence of beneficial bacterial depletion and overgrowth of opportunistic pathogens [27,28]. Meta-analyses have shown a reduction in alpha diversity, indicating impaired stability of the microbial network. Symbiotic bacteria that produce SCFA are generally depleted, including *Faecalibacterium*, *Roseburia*, and *Eubacterium* [29,30]. The metabolite of these bacteria, butyrate, is essential for maintaining intestinal barrier function and immune homeostasis [31]. At the same time, the abundance of potentially pro-inflammatory *Enterobacteriaceae* bacteria is significantly increased. Their abundance correlates positively with the severity of postural instability and gait disturbance (PIGD) in patients, suggesting that gut microbiota dysbiosis may directly contribute to the regulation of motor symptoms [26]. The abundance of *Akkermansia muciniphila* is commonly increased in PD patients, which may reflect compensatory changes in barrier function following mucus layer disruption [32].

The gut microbiota dysbiosis described above is not merely a compositional change; it also has far-reaching functional consequences. Together, these alterations form a potential bridge linking the gut to brain pathology. First, locally in the gut, the depletion of SCFA-producing bacteria reduces levels of key metabolites such as butyrate. This compromises intestinal barrier integrity, inducing a “leaky gut”, impairs energy metabolism in the intestinal epithelium, and suppresses the inhibition of local immunity [31]. Secondly, at the systemic level, circulating microbial metabolites and inflammatory mediators can trigger a low-grade systemic inflammatory state. By affecting BBB permeability, these factors may transmit inflammatory signals to the central nervous system [30]. Finally, at the neural level, gut microbiota dysbiosis can also directly or indirectly affect neurotransmitter balance and neuroinflammatory responses. This occurs through pathways such as modulation of tryptophan metabolism and the production of neuroactive substances, including γ-aminobutyric acid and dopamine precursors [33]. These findings suggest that the characteristics of the gut microbiota and their metabolite profiles may serve as biomarkers for the early diagnosis or staging of PD, and may also represent a potential target for intervention in the disease process [34,35]. These changes critically underpin the dysfunction of the gut–brain axis.

### 3.2. Proposed Involvement of the Gut–Brain Axis in PD Progression

The gut–brain axis is a bidirectional communication network connecting the gut and the brain. It encompasses neural pathways, such as the vagus nerve and the enteric nervous system (ENS), endocrine pathways (the hypothalamic–pituitary–adrenal axis), immune pathways, and metabolic pathways [36]. In PD, dysfunction of this axis is hypothesised to play a central role in the disease process [37]. The autonomous balance (vagal nerve tension), immune-inflammatory state and intestinal barrier function in these pathways are highly sensitive to exercise stimulation. This provides a theoretical basis for exercise to intervene in PD via the gut–brain axis [38].

The Braak hypothesis proposes that the pathological process of PD may begin in the gut [39]. Environmental factors trigger abnormal aggregation of *α-syn* in the ENS through a compromised intestinal barrier. These misfolded proteins then spread retrogradely via the vagus nerve, propagating in a “prion-like” manner to the brainstem and higher brain regions [40]. Multiple lines of evidence support this hypothesis. Vagotomy has been shown to reduce the risk of PD, and *α-syn* pathology has indeed been detected in the ENS of patients with PD [28]. Animal experiments have confirmed that *α-syn* originating from the gut can spread to the brain via the vagus nerve [41]. These findings highlight the vagus nerve as a key anatomical pathway linking intestinal pathology to central lesions. Notably, vagal tone is a key target modulated by exercise, particularly mind–body exercise [42]. In addition to the direct spread of *α-syn*, gut microbiota dysbiosis exacerbates PD pathology through multiple parallel mechanisms, collectively forming a self-perpetuating vicious cycle.

**Figure 1:**
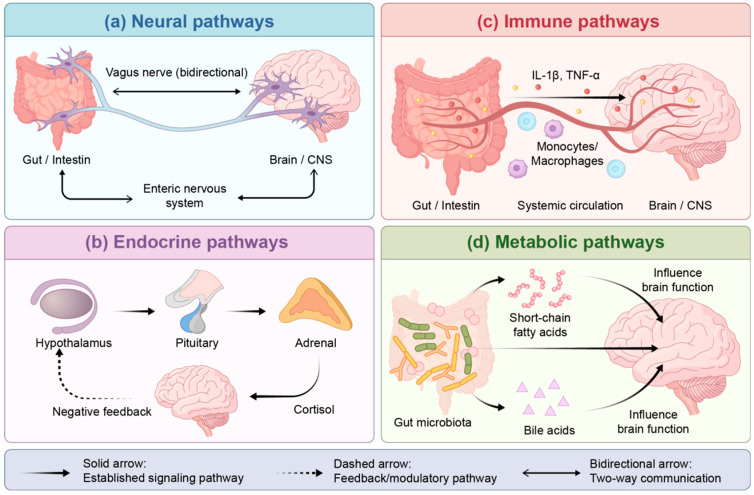
Schematic diagram of the gut–brain axis bidirectional communication network. (**a**) Neural pathways: Vagus nerve (bidirectional solid arrow) and ENS. (**b**) Endocrine pathways: Hypothalamic–pituitary–adrenal (HPA) axis and cortisol signalling (solid arrow) with negative feedback (dashed arrow). (**c**) Immune pathways: Cytokine-mediated communication (*IL-1β*, *TNF-α*) via systemic circulation. (**d**) Metabolic pathways: Microbial metabolites (SCFAs, bile acids) influencing brain function. Abbreviations: *IL-1β*, interleukin-1 beta; *TNF-α*, tumour necrosis factor-alpha. Note: Some pathways are supported by animal models or indirect evidence; the framework represents a mechanistic synthesis rather than fully established causality in PD patients.

Dysbiosis disrupts intestinal tight junctions, allowing bacterial products to translocate into the bloodstream, triggering low-grade systemic inflammation [43]. Circulating inflammatory factors can then cross the compromised BBB, activating microglia and amplifying neuroinflammation. The reduction in SCFA-producing bacteria leads to decreased levels of key metabolites such as butyrate, thereby weakening their protective regulatory effects on neurons and microglia. Dysbiosis dysregulates tryptophan metabolism, leading to an imbalance in the kynurenine pathway and promoting the accumulation of neurotoxic metabolites linked to depression and cognitive impairment in PD [44]. These three mechanisms are interconnected and mutually reinforcing. Importantly, each of these pathways is modifiable through exercise, as discussed in Section 4. As shown in Figure 1, the gut–brain axis achieves bidirectional communication through four pathways: neural, endocrine, immune, and metabolic.

### 3.3. Potential Mechanisms by Which the Gut Microbiota Regulates the Onset and Progression of PD

#### 3.3.1. Metabolic Mechanisms

The gut microbiota deeply regulates PD pathology through its metabolites. SCFA, bile acids, and tryptophan metabolites represent three core pathways.

SCFA, especially butyric acid, are present at lower concentrations in patients with PD, thereby compromising the integrity of the intestinal barrier. Butyric acid exerts neuroprotective effects through several well-defined mechanisms. As an inhibitor of histone deacetylases (*HDAC*), butyrate promotes the expression of anti-inflammatory genes [45]. As ligands for G protein-coupled receptors *FFAR2* (*GPR43*) and *FFAR3* (*GPR41*) [46], these receptors are expressed in intestinal epithelial cells, immune cells, and neurons. Butyrate can provide energy to intestinal epithelial cells, helping maintain the integrity of the barrier [47]. Furthermore, butyrate can cross the BBB and directly modulate microglial function, reducing their pro-inflammatory activation [48]. The depletion of SCFA-producing bacteria (such as *Faecalibacterium*) in patients with PD directly weakens the protective mechanisms described above [30]. Clinical studies have shown that for every 10% reduction in faecal butyrate levels in PD patients, the PIGD score increases by approximately 8% [49]. This suggests a dose–response relationship between microbial metabolites and the severity of motor symptoms. The gut microbiota metabolises primary bile acids to generate secondary bile acids. Among these, components such as ursodeoxycholic acid can activate the farnesoid X receptor *(FXR*) and the Takeda G protein-coupled receptor 5 (*TGR5)* receptor. This inhibits excessive microglial activation, reduces neuroinflammatory responses, and decreases neuronal apoptosis, thereby exerting important neuroprotective effects [50]. In patients with PD, the structure of the gut microbiota is significantly altered, leading to disturbances in secondary bile acid metabolism and abnormal changes in the bile acid profile [51]. This disturbance may weaken the protective signalling pathways mediated by *FXR* and *TGR5*, thereby exacerbating neuroinflammation and worsening neuronal damage [50].

In PD, tryptophan metabolism is often shifted towards the kynurenine pathway, leading to the accumulation of the neurotoxic metabolite quinolinic acid and a reduction in the neuroprotective metabolite kynurenic acid [52]. Quinolinic acid can activate N-methyl-D-aspartate (*NMDA*) receptors, induce excitotoxicity, exacerbate oxidative stress, and damage dopaminergic neurons [53]. Reduced levels of kynurenic acid, in turn, weaken endogenous protective mechanisms [52]. This metabolic imbalance not only exacerbates neuronal damage but may also form a vicious cycle with gut–brain axis dysfunction. Therefore, shifting tryptophan metabolism towards a protective direction is of significant value in slowing the progression of PD [52].

#### 3.3.2. Immune and Inflammatory Mechanisms

As the largest immune organ in the human body, the gut maintains a microbial homeostasis that directly regulates the inflammatory cascade from the local site to the central nervous system. This represents another core pathway by which the gut microbiota influences PD [54].

Disorders of the intestinal microbiota can trigger a cascade of effects, ranging from disruption of the intestinal barrier to systemic inflammation and, ultimately, neuroinflammation. This chain reaction involves lipopolysaccharides and other pathogen-associated molecular patterns (*PAMPs*), which activate Toll-like receptor 4 (*TLR4*) on peripheral immune cells, thereby mediating the transcription of pro-inflammatory cytokines (*TNF-α*, *IL-1β*, *IL-6*) through nuclear factor-κB (*NF-κB*). These cytokines can enter the central nervous system either through a damaged BBB or via activation of the vagus nerve afferent pathway. Once inside the CNS, these cytokines activate microglia and induce a shift toward a pro-inflammatory phenotype, characterised by excessive release of reactive oxygen species (ROS) and inflammatory mediators, thereby exacerbating dopaminergic neuron damage and promoting *α-syn* aggregation [55]. As detailed in Section 3.3.1, microbial metabolites such as SCFAs directly modulate microglial function. Beyond this metabolic pathway, gut microbiota dysbiosis also disrupts the balance between regulatory T cells (Tregs) and T helper 17 cells (Th17s), altering systemic immune tolerance and affecting the immune microenvironment of the CNS [56].

#### 3.3.3. Neural and Neuroendocrine Mechanisms

The gut microbiota communicates directly with the brain through neural and neuroendocrine pathways. Among these, the vagus nerve and the HPA axis are two core pathways that are highly sensitive to exercise stimulation [48].

The vagus nerve is considered the most direct physical connection. As the primary information highway between the gut and the brain, vagal afferent fibres can detect changes in the gut microbiota and its metabolites, thereby transmitting signals to the nucleus of the solitary tract. From there, the signals influence motor-regulating brain regions such as the substantia nigra and striatum [42]. Clinical evidence shows that vagotomy reduces the risk of PD development, highlighting the key role of this nerve in *α-syn* propagation. Vagal tone is an important target of exercise modulation, particularly for mind–body exercises such as Tai Chi [12]. By enhancing vagal efferent activity, exercise may in turn suppress intestinal inflammation and promote gut homeostasis, creating a bidirectional ‘gut–brain’ benefit [13].

The ENS serves as a local regulatory centre. In patients with PD, *α-syn* inclusions can be detected in enteric neurons, and intestinal dysfunction often precedes the onset of motor symptoms [57]. The gut microbiota influences intestinal motility and secretory function by modulating the balance of neurotransmitters in the ENS, including serotonin and GABA [33]. The clinical effect of exercise in improving constipation symptoms in PD patients may be partly attributable to its modulation of the ENS [58].

The HPA axis is central to the stress response. Gut microbiota dysbiosis and intestinal inflammation can activate the HPA axis, leading to persistently elevated cortisol and other glucocorticoid levels. Chronic high cortisol levels are neurotoxic and exacerbate non-motor symptoms such as depression and cognitive impairment [59]. Exercise is a recognised stress buffer that can optimise HPA axis function and reduce basal cortisol levels. This provides a neuroendocrine explanation for the beneficial effects of exercise on non-motor symptoms in PD [1].

#### 3.3.4. Temporal and Causal Relationships Between the Gut Microbiota and PD

In the onset and progression of PD, changes in the gut microbiota occur before, accompany, and dynamically influence *α-syn* pathology and the neurodegenerative process.

In PD animal models, dysbiotic features such as reduced gut microbiota diversity and depletion of SCFA-producing bacteria can often be detected before significant loss of dopaminergic neurons in the substantia nigra and before the onset of motor symptoms. This suggests that gut microbiota dysbiosis may occur at the preclinical stage and is not merely a passive consequence of neurodegeneration [60].

Harach and colleagues used germ-free mouse models to further reveal the driving role of gut microbiota dysbiosis. Mice that received faecal microbiota transplants from PD patients showed significantly earlier and more severe *α-syn* pathology in both the gut and the brain, compared with mice that received transplants from healthy controls. This evidence suggests that a specific dysbiotic microbiota could contribute to the initiation and progression of *α-syn* pathology. However, it remains unclear whether these microbiota changes are causal, contributory, or secondary to disease and treatment; the field has not yet reached consensus on this question [61].

Overall, gut microbiota imbalance occurring in early life or middle age may lower the brain’s resistance threshold to abnormal *α-syn* aggregation via the gut–brain axis. As the disease progresses, ongoing neuroinflammation and pathological changes in the central nervous system further worsen the gut environment, thereby accelerating the vicious cycle described in Section 3.2 [62].

**Figure 2:**
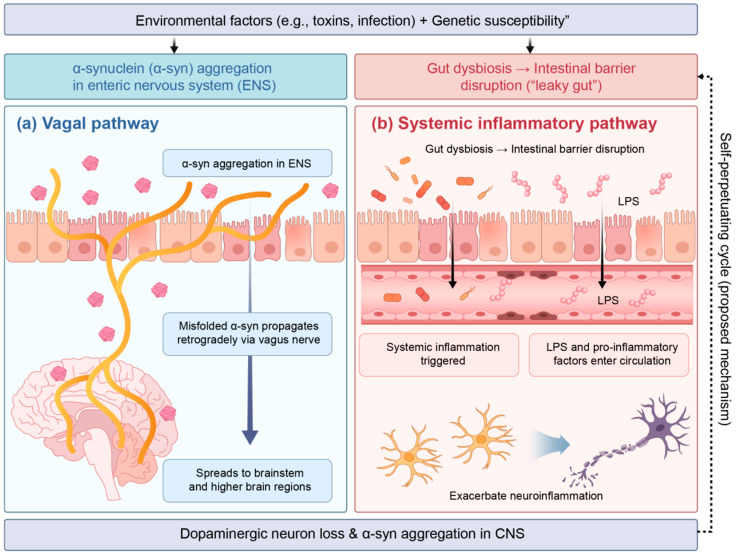
Gut microbiota regulate the pathological progression of PD through multiple pathways. Environmental factors and genetic susceptibility contribute to gut dysbiosis and intestinal barrier disruption, thereby triggering *α-syn* aggregation in the ENS. (**a**) Vagal pathway: Misfolded *α-syn* propagates retrogradely from ENS to the central nervous system (CNS) via the vagus nerve. (**b**) Systemic inflammatory pathway: Lipopolysaccharide (LPS) and pro-inflammatory factors enter circulation due to “leaky gut,” cross the BBB, activate microglia, and exacerbate neuroinflammation. The dashed feedback arrow indicates a self-perpetuating cycle (proposed mechanism). Note: Some pathways are supported by animal models or indirect evidence; the framework represents a mechanistic synthesis rather than fully established causality in PD patients.

This vicious cycle provides a critical temporal window for disease intervention [62]. Since a dysbiotic microbiota is sufficient to drive the pathological process, reversing or remodelling the microbiota should be an effective strategy to slow or even halt disease progression [63]. Among the various interventions available, exercise has become the most promising non-pharmacological therapy for “microbiota remodelling” due to its multi-target effects, low cost, high adherence, and well-established anti-inflammatory and pro-metabolic effects (see Figure 2 for a schematic representation of this cycle and the points of exercise intervention).

### 3.4. Dietary Regulation of the Gut–Brain Axis in PD

Diet is the most potent environmental factor shaping gut microbiota. High adherence to a Mediterranean diet is associated with an approximately 30% reduction in PD risk (RR = 0.71, 95% CI: 0.56–0.90) [64]. Dietary fibre intake of 10 g/d increment correlates with a 15% lower PD risk (HR = 0.85) [65].

Mechanistically, fibre is fermented into SCFAs (primarily butyrate), which strengthen the intestinal barrier, inhibit *HDAC*, and reduce microglial activation [31]. The Mediterranean diet also provides polyphenols and omega-3 fatty acids, which suppress *NF-κB*-mediated inflammation and may enhance the binding efficiency of exercise-induced myokines [66]. These diet-driven mechanisms operate independently and can synergise with exercise.

However, most dietary evidence in PD remains epidemiological or exploratory; large-scale RCTs are needed to establish causality and distinguish preventive from disease-modifying effects.

### 3.5. Bidirectional Role of Inflammation and Oxidative Stress in Gut Dysbiosis in PD

Inflammation and oxidative stress are not merely consequences of gut dysbiosis but also drivers that perpetuate it. In PD, elevated intestinal ROS and pro-inflammatory cytokines (e.g., *TNF-α*, *IL-1β*) favour the overgrowth of facultative anaerobes such as *Enterobacteriaceae* while suppressing obligate anaerobes including SCFA-producing bacteria [67].

Oxidative stress directly damages intestinal epithelial tight junctions, exacerbating “leaky gut” and promoting systemic translocation of bacterial products such as LPS [68]. This activates circulating immune cells and amplifies neuroinflammation via the vagus nerve and compromised BBB.

Exercise and dietary antioxidants (e.g., polyphenols) counteract this vicious cycle. Regular exercise upregulates endogenous antioxidant enzymes Superoxide Dismutase (*SOD*) and Glutathione Peroxidase (*GPx*) and reduces ROS production, while polyphenols scavenge free radicals directly [69]. Together, they help restore redox balance and suppress inflammation-driven dysbiosis.

Notably, the *NF-κB* pathway serves as a convergence point for inflammation and oxidative stress, and exercise suppresses its activation via *PGC*-1α-mediated antioxidant gene expression, while dietary polyphenols directly inhibit *NLRP3* inflammasome assembly [70].

## 4. Exercise Ameliorates PD by Modulating the Gut–Brain Axis

Regular exercise has become a core non-pharmacological intervention in the management of PD. Its benefits now extend beyond simple improvements in motor function to include potential disease-modifying effects [71]. Recent research has revealed that the neuroprotective effects of exercise are partly mediated by its multi-level regulation of the gut–brain axis [9,58]. This regulation follows a clear hierarchical structure. Exercise first remodels the gut microbiota ecosystem, then enhances intestinal barrier function, subsequently reduces peripheral and central inflammation, and ultimately promotes neuroplasticity [72]. Understanding this hierarchical cascade is key to elucidating the neuroprotective mechanisms of exercise.

### 4.1. Remodelling the Composition and Diversity of the Gut Microbiota

#### 4.1.1. Remodelling of Microbiota Diversity and Composition

The modulatory effects of exercise on the gut microbiota have been confirmed in multiple studies. However, these effects may differ between healthy individuals and PD patients, as well as among exercise modalities [11]. In general, regular exercise tends to increase the alpha diversity of the gut microbiota. This index reflects the stability and resilience of the microbial ecosystem, and PD patients are characterised by reduced alpha diversity [26].

At the level of microbial composition, exercise interventions show a selective remodelling trend that promotes beneficial bacteria while suppressing harmful ones [9]. Multiple studies, primarily in healthy populations and animal models, have reported that exercise can enrich SCFA-producing genera, including *Faecalibacterium prausnitzii*, *Roseburia*, and *Eubacterium* [9]. At the same time, exercise may inhibit the overgrowth of potentially pro-inflammatory bacteria, such as *Enterobacteriaceae*, thereby restoring gut microbial balance [72].

*Akkermansia muciniphila* has a dual role in the context of exercise and PD [73,74]. On the one hand, this bacterium helps maintain the integrity of the intestinal mucus layer, and its moderate increase is considered a beneficial adaptation to exercise; on the other hand, an abnormally increased abundance of *Akkermansia* is often observed in PD patients and correlates with disease severity [73]. This may reflect a compensatory response—an attempt by the body to repair itself by thickening the mucus layer after intestinal barrier damage [74]. Therefore, exercise-induced changes in *Akkermansia* should be understood as a restoration of homeostasis rather than a simple increase, and the effect depends on the baseline state [75].

#### 4.1.2. Differential Effects of Exercise Modalities

Research on the effects of different exercise modalities on the gut microbiota is still in its early stages. However, preliminary evidence suggests that differential effects exist [11]. Aerobic exercise has the most clearly established effects on increasing microbial diversity and enriching SCFA-producing bacteria [76]; There is less evidence regarding resistance training, and its effects may be partially mediated by myokines [77,78]; Mind–body exercises, represented by Tai Chi, may indirectly influence the gut microenvironment through the vagus nerve pathway, due to their modulatory effects on the autonomic nervous system. Diet is a major regulator of the gut microbiota, and the effects of exercise on the microbiota often interact with dietary intake [25]. Future studies need to control for dietary variables in their designs to isolate the independent effects of exercise [79].

#### 4.1.3. Coordinated Regulation of Intestinal Barrier and BBB

Intestinal barrier dysfunction, “leaky gut,” is a key link between gut microbiota dysbiosis and systemic inflammation in PD pathology [30]. Exercise has been shown to enhance intestinal barrier integrity through multiple mechanisms, among which the SCFA-mediated pathway is one of the best-characterised in preclinical studies [80]. Butyrate upregulates the expression of tight junction proteins, such as occludin, claudin, and zonula occludens-1 (*ZO-1*), by inhibiting *HDAC* and provides energy to intestinal epithelial cells, thereby strengthening the intestinal barrier [81]. Exercise reinforces this protective mechanism by enriching butyrate-producing bacteria (e.g., *Faecalibacterium*, *Roseburia*) [31]. Additionally, exercise enhances chemical and biological barrier functions by increasing intestinal mucus secretion and improving epithelial cell health. These effects collectively reduce the translocation of bacterial products (e.g., LPS) from the source, thereby blocking the initial step of gut-to-blood inflammatory signalling.

Beyond its effects on the intestinal barrier, exercise also protects the BBB through both direct and indirect pathways. Exercise-induced SCFAs (particularly butyrate) can cross the BBB. By inhibiting *HDAC* activity in cerebral vascular endothelial cells, they upregulate the expression of tight junction proteins and enhance the physical seal of the BBB [82]. Additionally, the shear stress generated by increased blood flow during exercise directly stimulates endothelial cells to release nitric oxide (NO) and other signalling molecules, thereby maintaining vascular health. Exercise promotes the expression of brain-derived neurotrophic factor (*BDNF*) and vascular endothelial growth factor (*VEGF*), which are essential for maintaining cerebral vascular endothelial function and microvascular integrity [81]. Exercise also improves systemic metabolic status and reduces systemic inflammation, thereby providing a favourable microenvironment for BBB stability [72].

The intestinal barrier and the BBB constitute two key lines of defence from the periphery to the central nervous system. Exercise regulates these two barriers in a coordinated manner, strengthening the intestinal barrier, which reduces the entry of pro-inflammatory stimuli into the bloodstream, thereby lowering systemic inflammatory load; in turn, stabilisation of the BBB further blocks the central penetration of any residual inflammatory signals [83]. This dual-barrier synergy may represent an important mechanistic basis for the central neuroprotection achieved by exercise through peripheral intervention.

### 4.2. Regulating Immune and Inflammatory Homeostasis

Exercise is a natural anti-inflammatory intervention [84]. Regular exercise can significantly reduce circulating levels of pro-inflammatory cytokines (*TNF-α*, *IL-1β*, *IL-6*) and increase the production of anti-inflammatory factors such as *IL-10*. This anti-inflammatory effect operates at three hierarchical levels: peripheral, central, and epigenetic.

During exercise, the cytokines released by contracting muscles play a central role [85]. *IL-6*, a pro-inflammatory cytokine at rest, exerts anti-inflammatory effects when acutely elevated after exercise by stimulating *IL-10* and inhibiting *TNF-α.* Additionally, by enhancing intestinal barrier function (as detailed in Section 4.1.2 and Section 4.1.3), exercise reduces the systemic inflammatory burden triggered by microbial translocation, thereby cutting off a major source of peripheral inflammation [54].

Persistent microglial activation is central to neuroinflammation in PD [56]. Exercise can induce a shift in microglia from a pro-inflammatory M1-like phenotype to a neuroprotective M2-like phenotype, enhancing their ability to clear abnormal *α-syn* and reducing the release of pro-inflammatory factors [86]. Exercise-induced SCFAs, particularly butyrate, partially mediate this phenotypic remodelling. Butyrate inhibits the activation of *NF-κB* by preventing its nuclear translocation and DNA-binding activity, and suppresses *NLRP3* inflammasome assembly and activation. Concurrently, SCFAs promote the metabolic transformation of microglia towards oxidative phosphorylation, driving these cells toward a homeostatic, anti-inflammatory phenotype. This metabolic-immune coupling provides a mechanism by which peripheral exercise signals are translated into central anti-inflammatory effects.

Recent research has revealed that exercise can induce “trained immunity” in innate immune cells. This is a form of non-specific immune memory that enables monocytes and macrophages to respond more precisely and appropriately to subsequent stimuli, rather than releasing excessive inflammatory factors. Regular moderate-intensity aerobic exercise—for example, three to five times per week at 60–75% of maximum heart rate—can shape a “low-response, high-homeostasis” innate immune phenotype through epigenetic reprogramming of haematopoietic progenitor cells in the bone marrow. This mechanism is important for controlling low-grade systemic inflammation and preventing the spark of inflammation from turning into a fire of neuroinflammation.

Taken together, the regulation of PD-related inflammation by exercise exhibits a clear hierarchical structure [58]. At the peripheral level, exercise suppresses the source of inflammation through myokines and by protecting the barrier. At the central level, exercise remodels microglial function through metabolic signals such as SCFAs [87]. At the epigenetic level, exercise reshapes the long-term response patterns of the innate immune system through trained immunity. These three layers of mechanisms work together to build an anti-inflammatory defence line from the gut to the brain. At the oxidative stress level, exercise upregulates the expression of *SOD* and *GPx* in both intestinal epithelium and brain tissue, reducing ROS-mediated damage to the intestinal barrier and dopaminergic neurons [88]. This antioxidant effect complements its anti-inflammatory actions.

### 4.3. Upregulating Neurotrophic Factor Expression and Promoting Neuroplasticity

Insufficient neurotrophic support, particularly reduced *BDNF* levels, is a key driver of neurodegeneration in PD [89]. Exercise efficiently elevates *BDNF* through three synergistic pathways, thereby counteracting neurodegeneration in PD. First, neuronal activity directly promotes *BDNF* transcription via *Ca^2+^/CREB* signalling. Second, muscles release myokines such as irisin, which activate the *CREB* pathway in the central nervous system [90]; third, SCFA produced by microbial metabolism, such as butyrate, relieve epigenetic inhibition of the *BDNF* gene [31]. This “neural–muscle–microbe” axis converts peripheral signals into central neurotrophic support, highlighting the multi-target advantage of exercise.

The exercise-induced elevation of *BDNF*, via the aforementioned three pathways (neuronal activity, myokines, and microbial metabolites), counteracts PD pathology through multiple mechanisms [90]. It promotes synaptic remodelling to compensate for dopaminergic deficits, enhances neuronal antioxidant and mitochondrial function, and supports neurogenesis [91]. Animal studies have confirmed that exercise improves motor function, increases dopamine release, and promotes neuronal survival in model animals, all of which are associated with elevated *BDNF* levels. Furthermore, exercise activates the *AMPK/PGC-1α* axis to promote mitochondrial biogenesis and enhances autophagy to clear *α-syn* aggregates [72]. The synergy of these multiple pathways builds a multi-layered neuroprotective network.

**Figure 3:**
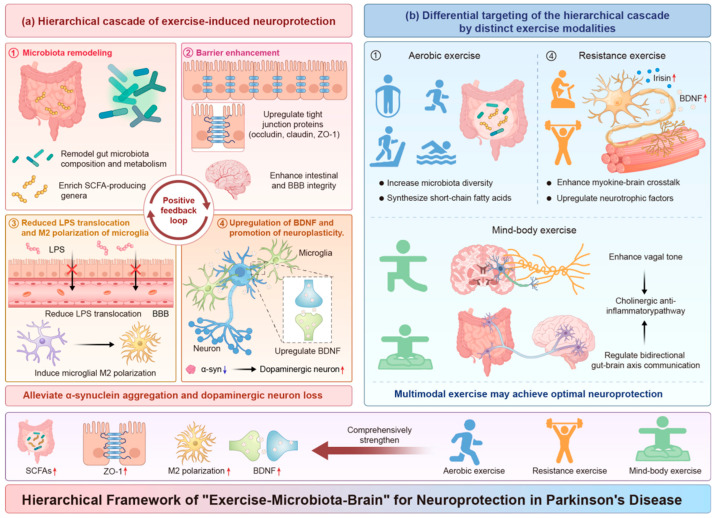
The “Exercise–Microbiota–Brain” hierarchical framework for neuroprotection in PD (**a**) Hierarchical cascade: Exercise remodels gut microbiota (①), enhances intestinal and BBB integrity (②), suppresses neuroinflammation (③), and upregulates neurotrophic factors (e.g., *BDNF*) to promote neuroplasticity (④). (**b**) Differential targeting by exercise modalities: Aerobic exercise primarily targets Level ① (microbiota remodelling). Resistance training targets Level ④ (*BDNF*) via myokines. Mind–body exercise (e.g., Tai Chi) may enhance vagal tone and potentially influence multiple levels. Symbols: ① Enrichment of SCFA-producing genera (e.g., *Faecalibacterium*, *Roseburia*); ② Upregulation of tight junction proteins (occludin, claudin, *ZO-1*); ③ Reduced LPS translocation and M2 polarisation of microglia; ④ Upregulation of *BDNF* and promotion of neuroplasticity. Note: Evidence for resistance and mind–body exercise is largely derived from non-PD populations or mechanistic hypotheses. Some pathways in this figure are supported by animal models or indirect evidence.

One of the important intervention potentials of exercise in PD lies in its ability to regulate *α-syn* pathology through multiple pathways [9]. The gut microbiota remodelled by exercise exerts its effects through multiple mechanisms: SCFA, particularly butyrate, enhance the autophagy-lysosome pathway and promote the clearance of *α-syn* aggregates [31]. They also reduce neuroinflammation, thereby weakening the environment in which pro-inflammatory factors induce *α-syn* aggregation [86]. As *HDAC* inhibitors, they epigenetically regulate the expression of *α-syn*-related genes, such as *SNCA* [87]. Furthermore, by strengthening the intestinal barrier and reducing intestinal inflammation, exercise may interfere with the upward propagation of *α-syn* pathology along the gut–brain axis. However, direct evidence in humans is currently lacking [86] (Figure 3).

### 4.4. Synergistic Effects of Exercise and Diet

Diet is the strongest environmental factor shaping the gut microbiota, accounting for approximately 20–30% of microbiota variation, a proportion that far exceeds the estimated contribution of exercise (approximately 5–10%) [92]. Exercise and diet may regulate gut–brain axis function through synergistic mechanisms [93,94].

Epidemiological studies have shown that for every 10 g/d increase in dietary fibre intake, the risk of PD is reduced by approximately 15% (HR = 0.85, 95% CI: 0.76–0.95) [95]. However, the clinical effects of dietary fibre supplementation alone in patients with PD have been inconsistent. A four-week prebiotic intervention study (fructo-oligosaccharides plus inulin, 10 g/d) found that while the abundance of Bifidobacteria in the faeces of PD patients increased significantly, no significant improvement in motor or non-motor symptoms was observed [89].

High adherence to the Mediterranean diet is associated with an approximately 30% reduction in PD risk (RR = 0.71, 95% CI: 0.56–0.90) [96]. In PD patients, a 12-week Mediterranean diet intervention significantly reduced the abundance of pro-inflammatory bacteria in faeces and decreased plasma TNF-α levels by approximately 25% [79].

From a mechanistic perspective, exercise and diet may converge on three common pathways. First, exercise enriches SCFA-producing genera while dietary fibre provides substrate, potentially amplifying butyrate levels [97]. Second, exercise reduces circulating *IL-6* and *TNF-α* via myokines, whereas the Mediterranean diet lowers the same cytokines via polyphenols [66]. Third, both exercise and dietary SCFAs upregulate tight junction proteins (occludin, claudin, *ZO-1*), reinforcing intestinal and BBB integrity through distinct but complementary mechanisms [98].

A conceptual model can be proposed: exercise serves as a “driver” that enriches beneficial microbiota and enhances vagal tone, while diet provides the “substrate” (fibre, polyphenols) and a “permissive environment” (reduced inflammation) that enables these microbial changes [99]. This synergy may be particularly relevant for interfering with the etiopathogenesis of PD, especially in early or prodromal stages.

It is important to note that not all neuroprotective effects of exercise can be attributed to the regulation of the gut–brain axis. Exercise also has a clear, independent effect distinct from that of the microbiota and can act directly on the central nervous system. Exercise directly upregulates brain-derived neurotrophic factor in the brain through mechanisms related to neuronal activity, and this process is independent of changes in the gut microbiota [100]. Exercise can increase blood flow and angiogenesis in the brain, providing direct vascular benefits [72]. Exercise improves overall metabolism and cardiovascular function, thereby indirectly promoting brain health [78]. Exercise reduces systemic oxidative stress by upregulating endogenous antioxidant enzymes.

Although these pathways unrelated to the microbiota are not the focus of this review, they undoubtedly play an important role in the overall neuroprotective effect of exercise. Therefore, the “exercise–microbiota–brain” framework proposed here should be regarded as one of multiple complementary mechanisms, rather than the sole pathway.

## 5. Analysis of Different Exercise Modalities in Alleviating PD by Regulating the Gut–Brain Axis

Different types of exercise, such as aerobic exercise, strength training, and mind–body exercises, may exert distinct yet complementary regulatory effects on the gut–brain axis. This is due to fundamental differences in their energy metabolic characteristics, neuromuscular recruitment patterns, and autonomic nervous system modulation [11]. Aerobic exercise is particularly effective at improving cardiorespiratory fitness and enhancing gut microbiota diversity. Resistance training regulates systemic metabolism through the secretion of myokines [77]. Mind–body exercise, with its unique capacity to modulate the autonomic nervous system, specifically enhances vagal tone, thereby directly influencing gut–brain communication [12]. Understanding these differences provides the theoretical basis for developing precision exercise prescriptions for PD [101]. This section will systematically compare the mechanistic evidence for how these three types of exercise intervene in PD through the gut–brain axis. The key differences between these three exercise modalities are summarised in Table 1.

### 5.1. Aerobic Exercise

Aerobic exercise (e.g., brisk walking, running, cycling, swimming) is the form of exercise with the most substantial evidence base for PD intervention [102]. Its regulation of the gut–brain axis begins with remodelling of the gut microbiota, proceeds through SCFA-mediated metabolic and immune signalling, and ultimately influences central neuroinflammation and neuroplasticity [9].

Long-term, regular moderate-intensity aerobic exercise can significantly increase the alpha diversity of the gut microbiota [58]. This index directly reflects the stability of the microbial ecosystem. As summarised in a recent review, clinical evidence suggests that aerobic exercise may increase butyrate-producing bacteria in patients with PD [58]. Butyric acid has dual protective effects at both the intestinal and central levels. These effects form a positive cycle with microbiota optimisation, while the systemic anti-inflammatory state induced by exercise, in turn, provides a favourable microenvironment for microbial homeostasis [78].

The dose–response relationship between aerobic exercise and microbiota modulation remains unclear. High-intensity interval training may produce different microbial effects compared with moderate-intensity continuous training. However, excessively high intensity carries a risk of exercise-induced gastrointestinal syndrome. The optimal weekly exercise volume for PD patients to obtain microbiota benefits, the duration of effects after exercise cessation, and the minimum effective intervention period all remain to be elucidated [11]. These gaps highlight the need for dose-finding studies to establish exercise prescriptions for PD.

### 5.2. Resistance Exercise

Resistance training—such as machine-based training, elastic band exercises, and bodyweight training—holds a unique position in the comprehensive management of PD [103]. Its core value lies in counteracting sarcopenia, progressive muscle weakness, and the resulting worsening of postural control impairment [104].

Resistance training is an effective physiological stimulus for inducing irisin secretion from skeletal muscle [77]. Irisin can cross the BBB and potently upregulate *BDNF* expression by activating the *CREB* signalling pathway [100]. *BDNF* plays a central role in supporting the survival, synaptic plasticity, and neurogenesis of dopaminergic neurons. This provides a molecular basis for the beneficial effects of resistance training on both motor and non-motor symptoms in PD [34].

Unlike aerobic exercise, the direct effects of resistance training on the gut microbiota remain inconsistent. However, it may indirectly modulate the gut microbial ecosystem through the following pathways [77]. First, through systemic metabolic optimisation. Resistance training increases muscle mass and improves insulin sensitivity and glucose homeostasis [78]. This optimised metabolic state may create an internal environment favourable for the growth of commensal bacteria. Second, through myokine–microbiota crosstalk. Emerging evidence, primarily from animal models, suggests that irisin may indirectly influence gut microbiota composition by modulating the function of intestinal immune cells. This “muscle–microbiota” axis represents a frontier in current research.

Currently, direct evidence for resistance training intervening in PD through the gut–brain axis comes primarily from animal models and healthy populations. Validation in PD patients remains lacking [103]. Resistance training should be regarded as a complementary strategy to aerobic exercise in the comprehensive management of PD, rather than a substitute [104].

### 5.3. Mind–Body Exercise

Tai Chi, Qigong, and Baduanjin, among others, as forms of physical and mental exercises, directly address the core movement deficiencies of postural instability and gait disorders in PD [12]. By integrating physical activity, breath control, and mental focus, these exercises may regulate gut–brain axis function through mechanisms that go beyond those of conventional exercise [12].

Through conscious breath control and mental focus, these exercises significantly enhance vagal tone, thereby directly modulating parasympathetic input to the ENS [105]. This “relaxation response” helps stabilise HPA axis function, lower cortisol levels, and reduce the negative impact of stress on intestinal barrier integrity and microbial homeostasis [94]. Slow, controlled movements optimise visceral blood perfusion and regular intestinal peristalsis, creating favourable conditions for the colonisation and metabolism of beneficial bacteria, without inducing the oxidative stress associated with high-intensity exercise. Evidence has shown that 12 weeks of Tai Chi practice can increase faecal SCFA levels and reduce pro-inflammatory cytokines in older adults [13], This suggests that mind–body exercise may exert indirect anti-inflammatory effects by modulating the metabolic function of the gut microbiota [93], However, it must be noted that these findings come from healthy older adult populations; direct evidence in PD patients is currently lacking. Circumstantial support comes from studies showing that Tai Chi improves constipation and autonomic function in PD [13], but mechanistic links to gut microbiota have not been directly tested.

Future studies should conduct head-to-head comparative trials to directly assess the effects of different types of mind–body exercise on gut–brain axis function in patients with PD. They should also explore the optimal combination strategies of mind–body exercise with aerobic exercise and resistance training.

## 6. Discussion

Although the aforementioned mechanism has constructed a coherent framework, some inconsistencies and limitations in the literature still require careful examination.

The issue of heterogeneity in research remains a major challenge. The PD patient population varies significantly in terms of disease stage (early vs. late), medication use (levodopa vs. no medication), and comorbidities. For instance, levodopa has been shown to directly alter the composition of the gut microbiota, potentially confounding analyses of disease-related dysbiosis [106].

It is common for human data to differ from animal data. The *MPTP* (1-methyl-4-phenyl-1,2,3,6-tetrahydropyridine)-induced PD mouse model reliably demonstrates that exercise can increase the abundance of bacteria that produce SCFA and reduce neuroinflammation [60]. However, corresponding human studies often show a smaller effect magnitude and greater individual differences. This may reflect differences in intestinal physiology, diet, or environmental exposure, which are controllable in animal studies but not in human trials.

The causal relationship of microbial community changes remains controversial. Although the Braak hypothesis and faecal microbiota transplantation experiments indicate that microbial imbalance can trigger pathological changes [49], longitudinal studies in individuals at high-risk stages are needed to track changes from the pre-exercise stage and determine whether changes in the microbial community occur before or after the onset of neurodegenerative diseases.

Biassed reporting of positive research results may exaggerate the consistency of exercise effects. Studies reporting no significant or negative results are published less frequently, which may lead to deviations in the assessment of relevant evidence.

### 6.1. Methodological Limitations of Existing Evidence

The gut–brain axis is merely one of the pathways through which exercise brings benefits. The neuroprotective effect of exercise results from the combined action of multiple pathways and cannot be attributed solely to changes in the gut microbiota [72]. Moreover, the direct benefits of exercise on the cardiovascular system, the direct stimulation of neural circuits, and the positive impact on psychological states are important pathways through which exercise improves PD symptoms [37]. The “exercise–microbiota–brain” framework proposed in this review should be understood as one of the core mechanisms of exercise benefits, rather than the only mechanism. This also highlights the unique value of exercise as a multi-target non-drug intervention—it acts on the nervous, metabolic, immune, and microbiological systems simultaneously, and this effect is difficult to achieve with any single drug.

Current research is unable to determine whether changes in the microbial community are the driving factor or the result of neurodegeneration in PD. Although the Braak hypothesis and studies on faecal microbiota transplantation support the former, longitudinal cohort studies on high-risk populations before the onset of PD are still needed to establish the temporal sequence between microbial community changes and the onset of pathology.

The methodological limitations of the current literature must be carefully considered. In terms of research design, most studies are small-sample cross-sectional studies lacking standardised microbial community sequencing protocols and uniform exercise intervention plans [72]. In terms of controlling confounding factors, factors such as diet, drugs, and comorbidities have not been adequately controlled, which may affect the reliability of the results.

### 6.2. Core Disputes, Future Directions, and Transformational Thinking

Currently, there are three major disputes in this field: whether the changes in gut microbiota are the driving factor or a concomitant phenomenon for the protection of motor nerves; whether the regulatory effects of different exercise methods on the microbiota are complementary or redundant; and whether there is a U-shaped curve relationship between exercise dose and the regulation of the gut–brain axis. In response to these disputes, future research needs to advance at three levels: establishing causal relationships through faecal microbiota transplantation experiments and integrating multi-omics technologies [6]; identifying the baseline characteristics of “exercise responders” and establishing precise exercise prescriptions; exploring the synergistic effects of multi-dimensional combined interventions such as “exercise + diet”. Regarding clinical transformation, precise exercise-prescription parameters cannot be provided at present. Based on existing evidence, moderate-intensity aerobic exercise shows potential to regulate the microbiota and reduce inflammation, and avoiding extremely high-intensity exercise may be a prudent approach. Early initiation of exercise intervention may maximise the neuroprotective effect, combining exercise with high-fibre or Mediterranean diets may enhance the efficacy, but these assumptions need to be verified through prospective trials. Clinical markers and microbial markers (such as faecal SCFA, abundance of faecal Bacteroides species) can be used as exploratory monitoring indicators, and their clinical utility needs to be verified [29].

### 6.3. Combined Exercise–Diet Interventions in PD

Despite strong mechanistic plausibility, high-quality randomised controlled trials testing combined exercise–diet interventions in PD patients are almost entirely absent [107]. Most existing studies examine exercise or diet in isolation, with dietary components often poorly controlled in exercise trials. Future studies should adopt factorial designs (e.g., exercise vs. no exercise; Mediterranean diet vs. usual diet) to quantify synergy [108]. Microbiome, metabolomics, and inflammatory endpoints should be included as mechanistic outcomes [107].

Whether exercise–diet synergy can truly interfere with the etiopathogenesis of PD—rather than merely alleviating symptoms—remains unproven in humans, but preclinical models and mechanistic reasoning support this possibility as a high-priority research frontier [107].

## 7. Conclusions and Perspectives

This review presents an “exercise–microbiota–brain” framework that summarises how exercise regulates the gut–brain axis in PD through a hierarchical cascade. It also shows that three types of exercise—aerobic, resistance, and mind–body training—achieve complementary effects through distinct pathways. The value of this framework lies not only in bringing together scattered mechanistic evidence, but also in offering a systematic way to understand the multi-target neuroprotective effects of exercise. Exercise acts on the microbial, immune, nervous, and metabolic systems at the same time—an advantage that no single drug can achieve.

Translating this framework into clinical practice will require two shifts in approach. First, move from a one-size-fits-all exercise prescription towards personalised plans based on the patient’s gut microbiota, disease stage and clinical features. Second, expand from exercise alone to combined exercise and nutrition strategies, making use of the strong influence of diet on the gut microbiota and the potential for synergy with exercise. Achieving this will depend on integrating multi-omics, wearable devices, and artificial intelligence to understand why individuals respond differently to exercise and to build data-driven models for precise prescribing.

As research on the gut–brain axis advances and evidence for non-drug interventions grows, precision lifestyle approaches based on the “exercise–microbiota–brain” axis are likely to move from theory to practice. This direction not only offers people with PD safer, more effective, and more sustainable personalised care, but also opens a new path for managing neurodegenerative diseases—targeting the gut to benefit the brain.

## Figures and Tables

**Table 1:** Comparison of mechanisms by which different exercise modalities regulate the gut microbiota to improve PD.

Comparison Dimension	Aerobic Exercise	Resistance Training	Mind–Body Exercise
Core target	Microbiota diversity, SCFAs	Myokines (irisin)	Vagal tone
Direct effect on microbiota	Clear (enriches SCFA-producing bacteria)	Inconsistent	Indirect (via autonomic nerves)
Main effector molecules	Butyrate, *BDNF*	Irisin, *BDNF*	Acetylcholine, SCFAs
Effect on barrier function	Clear (enhances intestinal barrier)	Indirect (via metabolic optimisation)	Clear (enhances vagal modulation)
Effect on neuroinflammation	Clear (inhibits microglia)	Indirect (via *BDNF*)	Clear (cholinergic anti-inflammatory pathway)
Strength of evidence (in PD patients)	Moderate	Low	Low to moderate
Suitable clinical stage	Early to middle	Middle to late	All stages, especially late

Note: Evidence strength is based on the number of available clinical studies, study design types (randomised controlled trials/observational studies/animal experiments), sample sizes, and depth of mechanistic validation. “Moderate” indicates that at least one high-quality randomised controlled trial in PD patients or several well-designed observational studies support the conclusion. “Low” indicates that evidence comes primarily from animal models, healthy population studies, or small-sample/cross-sectional studies in PD patients, and further validation in PD patients is needed. “Low to moderate” indicates a level between the two.

## Data Availability

No new data were created or analysed in this study. Data sharing is not applicable to this article.
